# A survey of relationship between anxiety, depression and duration of infertility

**DOI:** 10.1186/1472-6874-4-9

**Published:** 2004-11-06

**Authors:** Fatemeh Ramezanzadeh, Malek Mansour Aghssa, Nasrin Abedinia, Farid Zayeri, Navid Khanafshar, Mamak Shariat, Mina Jafarabadi

**Affiliations:** 1 Vali-e-Asr Reproductive Health Research Center,Gynecology and Obstetrics department, Tehran University of Medical Sciences, Imam Khomeini Hospital Complex, Keshavarz BLVD, Tehran 14194, Iran

## Abstract

**Background:**

A cross sectional study was designed to survey the relationship between anxiety/depression and duration/cause of infertility, in Vali-e-Asr Reproductive Health Research Center, Tehran, Iran.

**Methods:**

After obtaining their consents, 370 female patients with different infertility causes participated in, and data gathered by Beck Depression Inventory(BDI) and Cattle questionnaires for surveying anxiety and depression due to the duration of infertility. This was studied in relation to patients' age, educational level, socio-economic status and job (patients and their husbands).

**Results:**

Age range was 17–45 years and duration and cause of infertility was 1–20 years. This survey showed that 151 women (40.8%) had depression and 321 women (86.8%) had anxiety. Depression had a significant relation with cause of infertility, duration of infertility, educational level, and job of women. Anxiety had a significant relationship with duration of infertility and educational level, but not with cause of infertility, or job. Findings showed that anxiety and depression were most common after 4–6 years of infertility and especially severe depression could be found in those who had infertility for 7–9 years.

**Conclusions:**

Adequate attention to these patients psychologically and treating them properly, is of great importance for their mental health and will improve quality of their lives.

## Background

The impact of infertility on the psychological well being of couples involved has been the object of increasing attention in recent years. It cannot be denied that infertility is a deeply distressing experience for many couples [1]. In latter part of twentieth century, psychogenic cause was an accepted topic in infertility until when diagnostic abilities improved [2]. Edelmann et al (1985) found that infertility has a significant effect on psychological factors. Some authors have paid attention to the fact that health problems, loss of self-esteem, feeling akin to mourning, threat, sexual distress, depression, guilt, anxiety, frustration, emotional distress and marital problems are all associated with infertility [3].

We aimed to examine prevalence and severity of anxiety/depression in relation to duration/cause of infertility in Iranian infertile women.

Several studies have demonstrated anxiety has a detrimental effect on fertility [4] and that reduction of anxiety increases pregnancy rate [5,6]. Other researches failed to support a relationship between anxiety and infertility [7] Lapane et al (1995) indicated that depression could play an important role in the pathogenesis of infertility [8,9].

Infertility sometimes is accompanied by existential crises and emotional tensions such as anxiety, interpersonal problems, and suppressed anger, unsatisfactory in interpersonal, frustration, inferiority feeling, depression, rejected feeling and unconscious guilt feeling. Those couples with a history of failure in Assisted Reproductive Technique (ART) have shown personality maladjustment [10].

Overall percentage of psychological problem in infertile couples ranges between 25 and 60% [11]. One study has demonstrated that 74.6% patients reported changes in their mood [12]. Psychological difficulties of infertile patients are complex and influenced by a number of factors such as gender differences, cause and length of infertility. Freeman et al (1987) found that half of their sample of infertile couples described infertility as the most upsetting experience of their lives, whereas 80% of the sample reported by Mahlstedtet et al (1987) described their experience of infertility to be either stressful or very stressful [1]. Duration of infertility increases of stress [13]. Depression and anxiety were improved in infertile women as their age and duration of infertility increased [14]. Long lasting infertility and unsuccessful treatment cycles intensifies stress and psychopathologic problems especially depression [15,16]. Although, some studies showed that there is no relation between duration of infertility and depression or psychological factors [17].

Another study showed those who had 2–3 years infertility had more depression / anxiety than those who had this problem for a year or more than 6 years. Peak of depression could be seen during third year of infertility. After six years there will be a reduction in psychological symptoms in women. During first three years, infertility is accompanied by signs such as anxiety, depression, loss of self-esteem, impotence and maladjustment of marital status. After 3 years, optimistic attitude would change to despair and at last there will be some emotional changes to adopt a child or live without one, thereafter. Those who have social support, positive personal characteristics, and have a satisfactory life with their spouse show no signs of anxiety/depression [18].

Since most literature on psychological aspects of infertility is from developed countries it was thought that a study from a developing country with a different culture might contribute to existing knowledge on the topic. We aimed to examine prevalence and severity of anxiety/depression in relation to duration of infertility in Iranian infertile women. The results of this study, which included counseling and couple-therapy, are being prepared for infertile couples.

## Methods

The subjects were 370 infertile women who were referred to Vali-e-Asr Reproductive Health-Research Center between January 2001 and January 2002 for treatment of their infertility problems. In this group mean (± SD) age was 28 (± 5.37) and mean (± SD) duration of infertility was 6.36 (± 4.18). Among them 293 (79.2%) women were housewives and the others were working outside.

A gynecologist evaluated patients and then they were visited by a psychologist and were informed of the study purposes. Diagnosis of male factor is based on WHO criteria 1999. Menstrual history and mid-luteal progesterone level considers ovulatory factor, diagnosis of endometriosis is done by lapascopy, tubal factor is diagnosed by HSG and cervical facto is detected in Post Coital Test. After obtaining oral consent from each patient, data were collected using BDI [19,20], and Cattle [21]. inventories:

### Beck Depression Inventory (BDI)

The test used was a translated and validated Persian version of Beck's depression Inventory. A full 21-items BDI was administered. This scale is a widely used measure for intensity of depression.

Each item describes a specific behavioral manifestation of depression. Scores on each item can range from 0, indicating no depressive symptomatology, to 3, indicating a severe level of symptomatology. Total scale scores can thus range from 0 to 63. Scores of 17 or above it indicates of a clinically significant depression. The classification of depression scores involves:

1. 0–16 (without depression)

2. 17–27 (mild depression)

3. 28–34 (moderate depression)

4. 35–63 (severe depression)

### Cattle Inventory

The Cattle inventory is a 40 items self-report measure of anxiety. This test was a translated and validated Iranian version of Cattle's Inventory. Scores can range from 0 to 80, with scores of 28 or above demonstrate anxiety. Classification of anxiety scores involves:

1. 0–27 (without anxiety)

2. 28–40 (moderate anxiety)

3. 41–49 (neurotic anxiety)

4. 50–80 (severe anxiety)

For each patient the following data were recorded: age, cause and duration of infertility, education and job.

Data were analyzed by using statistical SPSS. The relationship between continuous and binary explanatory variables with Beck and Cattle scores were assessed using spearman's rho and unpaired t-test, respectively. In addition, the relationship between categorical responses and explanatory variables were evaluated using chi-square test. For descriptive purposes, we presented frequency tables.

## Results

Three hundred seventy infertile women were considered in this cross-sectional study. The results of Beck and Cattle inventories showed that 40.8% and 86.8% of these women had depression and anxiety symptoms, respectively (Table 1).

**Table 1:** Prevalence of depression and anxiety

Depression (Beck)	Frequency	Percent	Anxiety (Cattle)	Frequency	Percent
Normal	219	59.2%	Normal	49	13.2%
Mild	96	25.9%	Moderate	141	38.1%
Moderate	37	10%	Neurotic	117	31.6%
Severe	18	4.9%	Severe	63	17%
Total	370	100%	Total	370	100%

**Figure 1:**
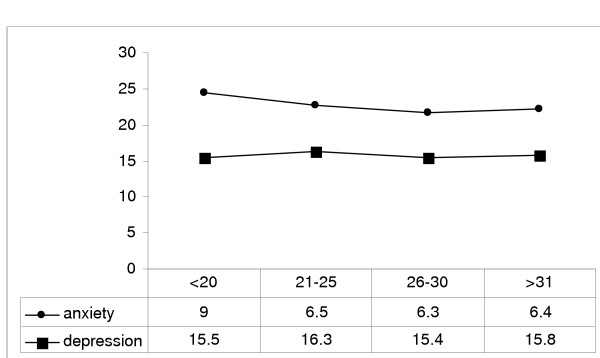
Mean score of depression/anxiety by age groups

Mean scores of depression and anxiety by age groups are shown in figure 1. Depression and anxiety were more severe in 21–25 years and under 20 years respectively, although there was no significant difference between age groups.

In next step, we compared the prevalence of depression (depression group is consisted of mild, moderate and severe depressive women) in all categories of infertility causes. Chi^2 ^statistic for this 2 × 9 table (chi-square = 20.643 P = 0.014) showed that prevalence of depression is not equal in these categories. Same analysis for the prevalence of anxiety (anxiety group is consisted of moderate, neurotic and severe anxietic women) in different groups of infertility causes showed no significant difference between them (chi-square = 7.491 P = 0.485) (Table 2).

**Table 2:** Frequency of depression and anxiety by cause of infertility

**Causes of Infertility**	**Percent of Depression**	**Percent of Anxiety**
Oligo-astheno-terato spermia	24.6%	86.2%
Azospermia	31.6%	80.7%
Ovulatory	48.0%	85.7%
Endometriosis	20.0%	80.0%
Uterus	52.2%	82.6%
Tubal	50.0%	90.5%
Habitual abortion	33.3%	100%
Unexplained	56.5%	95.7%
Male & Female (Both)	49.0%	93.9%
P-value	0.014	0.485

chi-square = 20.643

The correlation between Beck and Cattle scores based on Spearman's Rho was 0.707 (P < 0.001), which shows a significant relation between depression and/or anxiety scores. Then, we checked correlation coefficient between Beck and Cattle scores with quantitative variables like age, education (in years) and especially the duration of infertility.

Duration of infertility showed a significant relation with both Beck (r = 0.15, P = 0.004) and Cattle (r = 0.157, P = 0.002) scores. Based on duration of infertility 31(29.2%), 51 (42.1%), 30 (46.8%) and 39 (49.3%) patients had depression in different groups, but there was no significant relationship between duration of infertility and depression (P-value = 0.106) (Table 3).

**Table 3:** Frequency and rate of depression and anxiety based on infertility duration.

		**1–3 years**		**4–6 years**		**7–9 years**		**>10 years**		**All**	
		
		Freq.	Rate	Freq.	Rate	Freq.	Rate	Freq.	Rate	Freq.	Rate
**Depression stage (P = 0.106)**	**Normal**	75	70	70	57.8	34	53.8	40	50.6	219	59.2
	**Mild**	22	20.5	34	28.1	16	25	24	30.3	96	25.
	**Moderate**	5	4.6	12	9.9	8	12.5	12	15.1	37	10.
	**Severe**	4	3.7	5	4.1	6	9.3	3	3.7	18	4.8
**Anxiety Stage (P = 0.048)**	**Normal**	24	22.6	11	9	6	9.3	8	10.1	49	13.3
	**Moderate**	41	38.6	51	42.1	24	37.5	25	31.6	141	38.1
	**Neurotic**	29	27.3	36	29.7	24	37.5	28	35.4	117	31.6
	**Severe**	12	11.3	23	19	10	15.6	18	22.7	63	16.9

Also 82 (77.3%), 110 (90.9%), 58 (90.6%) and 71 (89.8%) of patients had different stages of anxiety and there was a significant relation between anxiety and duration of infertility (P-value = 0.048) (Table 3).

Educational level had a significant and negative relation with these two scores, but age showed no significant effect on depression and/ or anxiety (Table 4).

**Table 4:** Correlation between Beck and/or Cattle scores with duration of infertility, women's age and women's education

	**Beck scores**		**Cattle scores**	
	
	**Spearman's Rho**	**P- value**	**Spearman's Rho**	**P- value**
**Duration of Infertility**	0.150	0.004	0.157	0.002
**Women's Age**	-0.052	0.315	-0.044	0.395
**Women's Education (in years)**	-0.319	< 0.001	-0.156	0.003

Finally, we tested the relation between depression and/or anxiety scores with women's job. Results of unpaired t-test showed significant difference between depression scores of housewives and employees (t = 9.179, P = 0.003). Anxiety and depression were observed more in homemakers comparing to outside employees though we found no significant effect of women's job on Cattle scores (t = 2.943, P = 0.087) (Table 5).

**Table 5:** Relation between depression and/or anxiety scores and women's job

	**Women's job**	**Mean**	**SD**	**P-value**
**Beck**	**Housewife**	16.3549	10.2261	*0.003
	**Employee**	12.4286	9.7014	
**Cattle**	**Housewife**	6.3857	2.084	**0.087
	**Employee**	5.9091	2.4878	

*t = 9.179

**t = 2.943

## Discussion

Patients participating in this study were from different geographical areas in Iran. The finding of this study provides information about frequency and severity of anxiety/depression in order to duration of infertility in childless women.

The prevalence of psychiatric morbidity specially depression and/or anxiety in infertile patients have been assessed in several countries, for example Jones et al (1993) found that, there was mild to moderate depression in 28.3% of infertile women, moderate to severe depression in 7.2% and 1.2% had most severe depression based on BDI [7]. Another study showed that 67% of infertile women suffered from anxiety [1] and the same studied by Oddens et al (1999) reported that 24.9% had depressive disorders [22]. Anxiety were investigated in 130 infertile women in China, the results showed that different levels of mental pressure were found in 83.8% of infertile women, and moderate or severe types in 25% [23]. There was depression and/or anxiety disorder in 33% (Hong Kong), in 32 % (Scotland) of infertile women [16,22]. The overall percentage of depression disorder in infertile women ranges between 24 and 36% and also anxiety disorder ranges between 67 and 84%. Our study showed 40.8% depression and 86.8% anxiety in infertile women. Consistent with our research, Iranian infertile women show higher rates of anxiety and/or depression than the other countries. In Islamic and eastern countries such as Iran, family status especially childbearing is very important and valuable. Having a child stabilizes family and increases marital satisfaction. In our culture and society, negative attitudes to infertility are so throbbing. Having a child is psychologically or effectively, a vital factor for women, and the absence of children may cause marital problems such as divorce or even second marriage especially in Islamic societies which it is possible for men to marry with more than one woman. Intervention of relatives especially husband's family, negative attitude and behavior of surroundings (family, friends, neighbors, etc.) causes psychological problems for infertile women. Generally infertile women experience negative social consequences including marital instability, stigmatization and abuse. Infertility can have a serious effect on both psychological well being and social status of women in our country.

The most common age for depression and/or anxiety in our study was 21–25 years. In this study, anxiety and/or depression had negative correlation with education. In other words, with the raise of education level, anxiety and/or depression decrease.

Results of different studies about relationship of age and education with anxiety and/or depression were not similar. Age and education level have no significant relationship with depression and/or anxiety [24]. Another study showed that there was positive correlation between them [18]. In such closed societies as some parts of our country, education and job may be the lone gate leading women to joyful aspects of their life other than maternity. This is why education plays a considerable role in decreasing their depression/anxiety.

Having a job may reduce stress from In Vitro fertilization (IVF) [25]. In our study, anxiety and/or depression were observed more in housewives (vs. outside employees). It seems being at work outside home decreases psychological signs of anxiety and depression.

Based on previous researches [26-28], infertile women showed higher rates of psychiatric symptoms than their partners, especially in female and unexplained factors. Women are necessarily more deeply involved in treatment procedures and it is normal for them to be more affected. One of the characteristics of infertile couples is that women are habitually more affected by the situation of infertility than men [29].

Based on our study depression is more common in "unexplained cause" group comparing to other causes and anxiety is more common in "endometriosis" group comparing to other causes, our results is similar to other studies [2,3,6,9,13].

Anxiety and/or depression increases with duration of infertility [30]. A study demonstrated that women who had experienced infertility for a long or medium range of time presented a significantly lower state of anxiety [31] and there was a trend of decreasing psychological stress with lengthening of infertility time. Based on depression scales, infertile patients who had infertility for an intermediate to a long time showed less symptoms than those who are in their first stage of their problem [32] but other studies showed that psychological distress in infertile women increase with time [15] and depression peaks between the second and third year of infertility and does not return to normal range until after 6 years of infertility [18]. In our study, women with lower stages of depression and anxiety can be seen during 1–3 years of infertility, but during 4 through 6 years after infertility diagnosis their signs become more prominent, especially severe depression has the most common frequency during 7–9 years. It was shown that the first three years (1–3) anxiety and/or depression is in its lowest limit and after 4 to 9 years it becomes worse. It seems that our results are completely different comparing to other countries. It may show that having a child is very important for our people, especially our women, therefore women show higher and longer emotional reactions and psychiatric symptoms lasts longer in comparison to other countries.

In conclusion it can be suggested that psychological interventions especially in 4–9 years of infertility may prevent the surge in depression/anxiety and could presumably lead to increased pregnancy rates.

## Authors' contribution

FM contributed to design and management of the study.

NA contributed to study design and data gathering.

The initial idea of this study was by MMA.

NK contributed in design, writing of the manuscript and analysis of this research.

FZ contributed in design, writing of the manuscript and analysis of this research.

MS contributed in edition and revision of the article.

MJ contributed in edition and revision of the article.

## Pre-publication history

The pre-publication history for this paper can be accessed here:


